# Comparison of MTA, CEM Cement, and Biodentine as Coronal Plug during Internal Bleaching: An In Vitro Study

**DOI:** 10.1155/2020/8896740

**Published:** 2020-11-12

**Authors:** Paridokht Zarean, Parichehr Zarean, Arash Ravaghi, Maryam Zare Jahromi, Mitra Sadrameli

**Affiliations:** ^1^Dental Implant Research Center, Dental Research Institute, Isfahan University of Medical Sciences, Isfahan, Iran; ^2^Isfahan (Khorasgan) Branch, Islamic Azad University, Isfahan, Iran; ^3^Department of Endodontics, Isfahan (Khorasgan) Branch, Islamic Azad University, Isfahan, Iran; ^4^Private Practice, Chicago, Illinois, USA; ^5^University of Washington, Seattle, Washington, USA

## Abstract

**Background:**

Internal bleaching is a choice of treatment in discolored endodontically treated teeth. Cervical root resorption is one of the important complications of this treatment. A suggested procedure to prevent this type of resorption is using a coronal barrier under the bleaching materials. The aim of the study was to compare the microleakage of mineral trioxide aggregate (MTA), calcium-enriched mixture (CEM) cement, and Biodentine.

**Materials and Methods:**

In this in vitro study, a total of 60 single canal incisors were included. They were randomly divided into three experimental groups (*n* = 16), one positive control group (*n* = 6), and one negative control group (*n* = 6). Coronal portion of the canals in the experimental groups was sealed with 3 mm of MTA, CEM cement, or Biodentine as a coronal barrier. After 3 days, specimens were bleached. A fresh *Enterococcus faecalis* suspension was added to the samples. The culture tubes were observed for 45 days, and the daily turbidity was recorded. Statistical analysis was accomplished by the Kaplan–Meier test and SPSS 22.

**Results:**

All positive samples showed turbidity, whereas none of the negative samples allowed bacterial leakage. Results showed no significant difference between MTA, CEM cement, and Biodentine groups. (*P* value = 0.304, 0.695, and 0.217). The bacterial microleakage for the two groups also did not show significant differences.

**Conclusions:**

CEM cement and Biodentine showed promising results as coronal plug, and clinical studies are needed to test these materials with MTA for avoiding microleakage in internal bleaching treatment.

## 1. Introduction

Internal bleaching is a method commonly used in discolored teeth after root canal treatment. Bleaching materials increase the osteoclastic activity and produce undesirable effects such as necrosis of the cementum and periodontal inflammation, which may result in root resorption [1–3]. Regarding periodontal inflammation, we have to consider other factors such as periodontal bacteria as well as antibacterial activity of nutraceutical agents [4–6]. In the cases where the gutta-percha lacked a protective layer, the occurrence of cervical resorption has been reported with a range of 1 to 13% [7]. Fuss et al. [8] measured the pH in areas surrounding teeth with pulp chambers filled with bleaching material and reported leakage to the external periradicular environment teeth.

Ideally, root canal filling material should be effective as a barrier in preventing the passage of material and bacteria from the oral cavity through the canal into periapical tissues, but none of the recent obturation materials or techniques have been able to successfully create such a blocking function [9, 10]. This issue has led to the insertion of an intraorifice plug with a thickness of at least 2 mm on the pulpal orifice after completion of orthograde root canal treatment [11].

The importance of the coronal plug has been extensively studied and reported that with an appropriate cervical seal, even 20 years after the tooth whitening, no cervical resorption has been observed [12]. For this purpose, various materials such as amalgam, zonalin, zinc phosphate cement, zinc oxide eugenol, resin composite, dentin coatings, glass ionomer (GI), mineral trioxide aggregate (MTA), calcium-enriched mixture cement (CEM cement), and Biodentine as coverage for the orifice of the canals during the whitening process has been proposed. The recommended standard protective plug is GI cement, which can remain as the base for the final filling [7].

Many studies have shown that MTA, due to its microleakage resistance, high marginal adaptation, and high concentrations of calcium hydroxide, is a suitable material to use as a plug to prevent root cervical resorptions. It is assumed that MTA prevents cervical resorptions due to its alkaline properties [13–18]. However, the potential for color change, the presence of toxic elements in the composition of the material, the difficult application procedure, long setting time of 165 ± 5 minutes, high cost, lack of solubility, and difficulty in removing the set material are among its disadvantages [19, 20]. CEM is another biomaterial cement, a hydrophilic and homochromatic cement, that has alkaline and antibacterial properties [21]. CEM cement combines MTA biocompatibility and more efficient features, including shorter setting time, better applicable properties, and no posttreatment dental discoloration. In addition, CEM cement is simultaneously able to induce the formation of hard tissue as well as an effective seal. The higher fluidity of the CEM cement makes its thickness less than MTA [22]. Currently, calcium silicate-based materials, due to their similarity to MTA in composition and biologic properties as well as similar clinical properties, are also very popular. Meanwhile, Biodentine is a compound cement with mechanical properties similar to those of dentin [23]. Studies have shown that Biodentine has a better ability to seal, higher compression strength, shorter setting time (12 minutes), decreased microleakage, less color change, and better antimicrobial properties. Biodentine also has fewer toxic effects and greater biocompatibility than MTA [24–26]. The disadvantages include the potential of tooth discoloration and high cost of the material [27]. There are different methods to assess the leakage during intracoronal bleaching from dye penetration and fluid filtration to chemical and microbial tests [11].

The purpose of this study was to compare the microbacterial leakage prevention of MTA, CEM cement, and Biodentine as coronal plugs during internal bleaching treatment in endodontically treated teeth. The null hypothesis is that there is no statistically significant relationship between MTA, CEM cement, and Biodentine as coronal plugs.

## 2. Materials and Methods

For this in vitro study, 100 extracted central and lateral permanent single-root human teeth from multiple clinics in Isfahan were collected and were stored in normal saline. As part of the final evaluation, periapical radiographs were taken. The exclusion criteria included teeth with more than one canal, fractured and carious teeth, and those with immature, resorbed, or curved apices. Sixty teeth were selected. Samples were placed in a solution of sodium hypochlorite (5.25%) (Golrang Industrial Group, Tehran, Iran) for 5 minutes. The remaining external debris on the surface of the teeth was completely removed by scaling and brushing. Before the experiment, the extracted teeth were stored in saline solution (sodium chloride 0.9%).

Numbers of samples are calculated according to the following formula:

(1) $$\begin{array}{c} n=\frac{{\left(Z_{1-\left(\alpha /2\right)}+Z_{1-\beta }\right)}^{2}\left(\sigma_{1}^{2}+\sigma_{1}^{2}\right)}{d^{2}} \end{array}$$

According to the previous studies: *σ* = 0.46, *α* = 0.05⟶*z*_1−(*α*/2)_ = 1.96, and 1 − *β* = 0.80⟶*Z*_1−*β*_ = 0.84.

Among the prepared samples, 6 teeth were blindly pulled out of a container to serve as the negative control group. In the remaining samples, access cavities were created by using a high-speed handpiece with water coolant fissure bur no. 1, and the pulp horns were removed by turbine round bur no. 1.4 (Tees Kavan Company, Tehran, Iran). After determining the length of the canal with a #10 K-file, the working length was determined by subtracting 1 mm from the canal length using periapical radiographs. The root canals were instrumented by step-back technique to a #35 K-file (Mani, Japan) up to 3 mm shorter than working length. The coronal portions were flared by Gates–Glidden nos. 1, 2, and 3 (Mani, Japan).

During all stages of preparation, canals were washed with 2 cc sodium hypochlorite solution 2.5%. The specimens were randomly selected and consecutively assigned to 3 experimental groups of MTA (*n* = 16), CEM cement (*n* = 16), and Biodentine (*n* = 16) and a positive control group (*n* = 6) and encoded in separate containers. The prepared canals were obturated by using the lateral condensation method, and access cavities were sealed with a small cotton pellet and Cavit (Cavisol, Golchai, Iran). To ensure the obturation quality of the canals, periapical radiographs were taken. With the exception of the positive control group in which no materials were placed on the gutta-percha and the Cavit remained intact, after one week, to allow enough setting of the obturation sealer (AH26 Sealer, Dentsply, Germany), the Cavit was removed in the 3 experimental groups [28]. Two millimeters of gutta-percha from the level of the CEJ was removed by peeso reamer, and depth of the created space was measured using a periodontal probe and was cleaned by 70% alcohol-moistened microbrushes followed by applying resin to block dentinal tubules. The thickness of at least three mm from the MTA bases (Angelus Company, Londrina, PR, Brazil), CEM cement (Yekta Zist Dandan Company, Tehran, Iran), and Biodentine^TM^ (Septodent, St. Maur-des-Fossés, France) were individually placed in the orifice with greater thickness in the labial and lingual surfaces and less thickness in the proximal areas to follow the ideal vertical outline of the protective layer which should be 1 mm coronally to the CEJ [29–31].

Each experimental group was prepared according to the manufacturer's instructions, so the MTA was mixed with a 3 to 1 powder to liquid ratio on a glass slab for optimum consistency. The powder and liquid of CEM cement were also mixed on a glass slab to obtain appropriate consistency. Five drops of Biodentine liquid was added to a capsule of powder which was amalgamated with 4000 rpm for 30 seconds. Materials were carried to the intraorifice cavity by the end of a wet paper cone and finally compressed with an endodontic plugger. Wet cotton pads were placed on MTAs, and the teeth were dressed by Cavit.

After 72 hours in which the initial and final setting of the access cavity plug materials were completed, the dressing of all 54 teeth was completely cleaned and replaced with a mix of sodium perborate paste (SD Fine-Chem L, India) and physiological serum [7]. In order to remove the excess moisture, sodium perborate paste was packed and sealed with a fine cotton pad and Cavit. The process of bleaching was carried out three times in a 72-hour interval [7, 29]. After this, the access cavity was completely cleaned. To prevent bacterial leakage from the lateral canals and cement rupture areas, with the exception of the apices and the access cavities, 3 layers of nail varnish were applied to the root surfaces. This was done for the 3 experimental as well as the positive control group. In the negative control group, the entire root except the access cavity was covered by 3 layers of nail polish. Each group received a different nail polish color [32].

**Figure 1:**
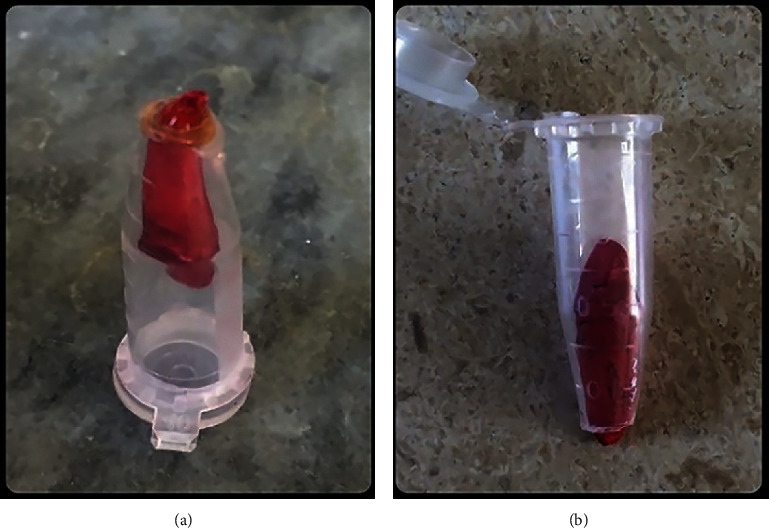
Sealing the gap between sample and tube.

In order to assess the bacterial microleakage test, the teeth were transferred to a system that has two upper and lower lacunas. The upper lacuna was made by cutting off the 5 mm end of the Eppendorf tubes (Padtan Teb, Tehran, Iran). The specimens were passed through the tube until they just passed the bottom of the tube. The distance between each root and tube was sealed with cyanoacrylate adhesive (Bonfix, Isfahan, Iran) and Hoffman (Hoffmann Dental Manufaktur GmbH, Berlin, Germany) to prevent microleakage (Figure 1). This connected model of tube and tooth was sterilized by use of an ethylene oxide autoclave for 8 hours.

**Figure 2:**
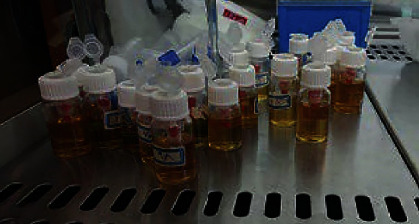
Compressing the upper lacuna (included samples) into lower lacuna (included medium) under sterile conditions until 2-3 mm of the apical areas were placed in BHIB medium.

We inserted a volume of about 8 to 10 ml of sterile brain-heart-infusion broth (Hi-Media, Germany) into disposable cultural tubes. These tubes containing the medium were used as the lower lacuna of the microleak test system. The upper lacunas with teeth were compressed into the lower lacuna, under the aseptic conditions under a chemical hood, until 2-3 mm of the apical areas were placed in the BHIB medium (Figure 2).

The connection areas between the upper and lower lacunas were closed with the help of parafilm strips. To ensure sterilization, the whole system was incubated aerobically at 37°C for three days. Any sample, which showed evidence of turbidity in the medium, was further excluded from the study.

**Figure 3:**
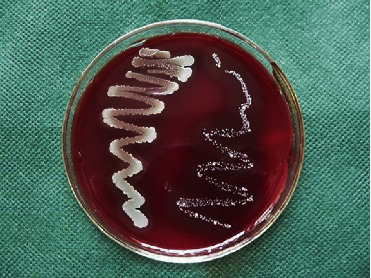
Culturing turbidity solutions in blood agar medium.

2 ml of BHIB medium with a count of 9 × 10^8^ CFU/ml *E. faecalis* ATCC 29212 (collection center of fungi and industrial bacteria in Iran) was mixed to form a fresh bacterial suspension. 100 *μ*L of this suspension was applied to the upper lacuna of the microleakage test system every two days. The previous suspension was washed by phosphate-buffered saline (PBS), and the fresh suspension was applied for the next two days [32]. Bacterial microleakage was evaluated by the formation of turbidity in a lower BHIB medium [33]. Samples were examined daily for 45 days. Once the turbidity was detected in each sample, the time of occurrence was recorded and the sample was excluded. The turbidity solution of each sample was cultured in a medium of blood agar to ensure that the contamination agent, according to iridescent color and special morphology of colonies, was only *E. faecalis* bacterium (Figure 3).

Data were analyzed by SPSS 22 software (IBM Co, Chicago, IL, USA), and survival analysis was performed by use of the Kaplan–Meier method (significant difference was considered as *P* < 0.05). This research was approved by the Ethics Committee of Islamic Azad University, Isfahan (Khorasgan) branch (no. 23810201902002).

## 3. Results

The results showed all positive group samples had complete bacterial microleakage on the first day. However, by the end of the experiment, none of the negative control samples showed signs of bacterial microleakage. The results from the control groups indicate the validity of the study. Bacterial microleakage was observed in 11 out of 16 samples in the MTA group (31.25% without turbidity), 12 out of 16 samples in the Biodentine group (25% without turbidity), and 9 out of 16 samples in the CEM cement group (43.75% without turbidity).

The significance of duration of bacterial microleakage in study groups, using the Kaplan–Meier method (Log Rank), showed that there were no significant differences between the bacterial microleakage of MTA, Biodentine, and CEM cement. In comparing two experimental groups, there was also no significant difference in survival time between MTA and Biodentine (*P* value =0.304), MTA, and CEM cement (*P* value =0.695), Biodentine and CEM cement (*P* value =0.217).

The mean and median survival time for microleakage as a valid index at 95% confidence interval in Table 1 shows that CEM cements resisted more days against leakage and preserve better seal over time. The median time of microleakage was lowest in Biodentine and, in less days, 50% of the samples had microleakage and lost proper seal. Although there are apparent differences in the mean numbers obtained from the Biodentine and other groups, there was no significant difference in their bacterial microleakage resistances (*P* value =0.304 and 0.217).

**Figure 4:**
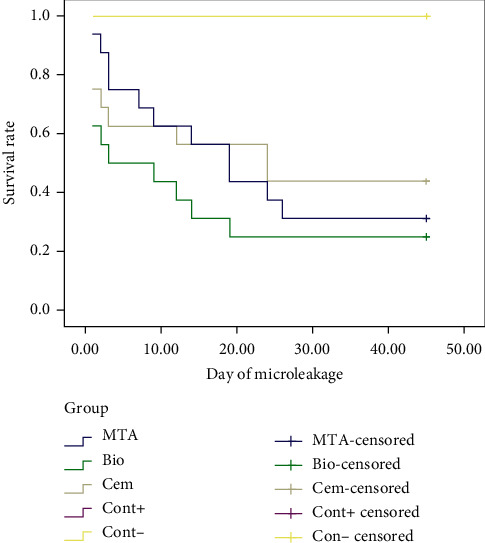
Rate of microleakage over time in experimental groups (338 × 190 mm) (96 × 96 DPI).

**Figure 5:**
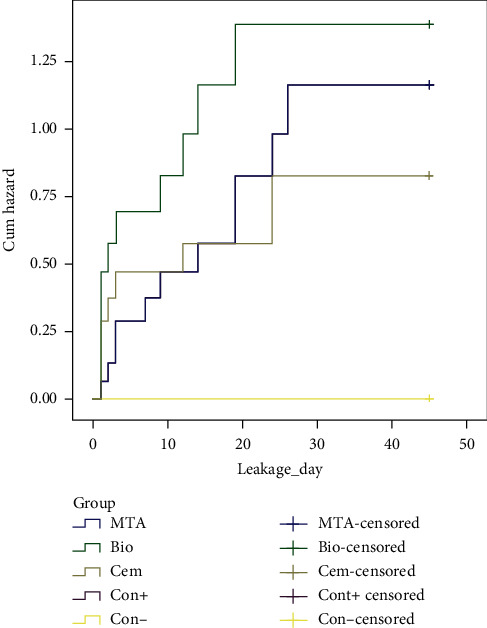
Hazard function.

The highest rate of microleakage among three experimental groups in first days was related to the Biodentine group. In terms of the rate of microleakage occurring over time, the results showed that the MTA group has a steeper slope and is expected to present a lower survival rate over a longer period of time (Figures 4 and 5).

## 4. Discussion

An aesthetic issue in endodontically treated anterior teeth is discoloration for which patients often seek further treatment. Internal bleaching is one of the most common techniques of whitening for nonvital teeth. But, this method sometimes has led to adverse effects such as cervical root resorption, which is one of the most destructive consequences. A proposed method to prevent this resorption is use of the coronal plug in the orifice of the root canal [1, 2].

The proper bond strength between the materials used as a barrier and root dentin has a significant role in preventing cervical resorption after bleaching. Considering the fact that different coronal plugs may have different effects in preventing cervical root resorption, the main aim of this study is to compare the microleakage of MTA, CEM cement, and Biodentine, since the composition of all three materials can produce hydroxyapatite crystals and prevent microleakage [34]. In previous studies, these three materials were not compared with each other using the bacterial microleakage test.

Amongst the different methods proposed to evaluate the sealing ability of apical barriers, color penetration is one of the oldest. Since the chemical properties such as the pH and the size of the molecules, as well as their ability to discolor materials such as CEM cement, MTA, and calcium hydroxide, can influence the amount of penetration and ultimately the results, it is reasonable to use alternative methods for assessing the degree of microleakage [35]. The use of bacteria to assess microleakage in in vitro studies is safe and comparable to clinical conditions [33].

Other studies have used different bacterial species such as anaerobic streptococci related to *Peptostreptococcus micros* and *Prevotella intermedia*, which may have led to their contradictory results [36]. Therefore, in the present study, *E. faecalis* was used to simulate oral conditions. It is a normal oral flora, able to withstand adverse environmental conditions as well as penetrates into dentinal tubules, and is the most common species cultured from failed root canals [30, 32, 37]. Size of bacteria and being aerobic or nonaerobic are two criteria which may lead to different results.

Several studies have also evaluated the antibacterial properties of MTA, CEM cement, and Biodentine and have concluded that although these substances owe their significant antibacterial properties to high pH, neither of them has been able to remove *E. faecalis* [38, 39].

On the other hand, some studies showed that MTA, CEM cement, and Biodentine have antibacterial effect on *E. faecalis*, but different studies showed different significance [40, 41]. So, in this study, we consider antibacterial effects for all coronal plugs without any significant differences which may be present.

Based on the present study, there was no significant difference in the mean amount of microleakage between the experimental MTA and CEM cement groups. This result is consistent with the results of the study by Zare Nezhad et al. [7]. However, in these studies, MTA has shown better results than CEM cements. The difference in the number of specimens, the type of tooth, the time and design of the test, and the method of assessing the microleakage may be helpful in explaining the difference in results.

Declining the microleakage of CEM cement can be attributed to the higher flux of this material and its better adaptation with dentinal walls [42]. It can be said that one of the reasons for MTA's greater microleakage compared to the CEM cement is the higher viscosity of MTA, which can result in a decreased adaptation with the dentinal walls and flowing reduction [43].

In addition, Moghadam et al. [44] measured the microleakage of MTA and CEM cement as coronal plugs in internal bleaching by the color penetration method and reported no significant difference in the microleakage between these two groups. However, the average of the microleakage in the CEM cement group was lower than the MTA group, which is consistent with current study results.

In another study by Ramezani and Sadeghi [45], comparing the bacterial microleakage of CEM cement and MTA as a filling material of furcation in deciduous teeth, the authors reported the same results in three experimental groups that were consistent with the results of the present study. In their studies, the lowest microleakage and the highest microleakage were reported in MTA and Biodentine groups, respectively. In the present study, the highest microleakage was observed in the Biodentine group, although the lowest microleakage was related to the CEM cement group, which was different from the results of Ramezani and Sadeghi. It is possible to consider the type of tooth, the thickness, and location of the material, and the manner in which the tests were performed as potential explanations for these differences.

In the study of Bolhari et al. [46], there was no significant difference between marginal adaptation of MTA, CEM cement, Biodentine, and bioaggregate, using electronic microscope scanning. Their results showed that, in the presence of blood, the maximum gap was in Biodentine, and the minimal gap belonged to CEM cement, which further corresponds to the results of this study.

In the presence of normal saline, the maximum gap was related to CEM cement and the lowest gap was related to Biodentine [46]. In studies that are performed by electronic microscope scanning, there is a need to cut the teeth that may make crack or change in the microscopic view between the dentin of the root and the repairing materials. Another problem of this method is that the studies are carried out on two-dimensional images that depend on the cutoff cross-sections. While in two-dimensional images they may present with potential areas of leakage, they may present with adequate seal in three-dimensional images. In our opinion, this explains the differences in their conclusion with the present study.

In a similar study, Ramezanali et al. [47] measured the microleakage of Biodentine, MTA, and CEM cements as coronal plugs by use of the color leakage method. They also noted no significant differences in the microleakage of the three different materials. The lowest microleakage was observed in the CEM cement group, which is consistent with the present study, and the highest microleakage was observed in the MTA group. This difference can be due to the differences in the measurement methods. The color-based microleakage method poses unique problems, including the effect on the chemical properties and the pH of the material being tested, such as the possibility of neutralizing the small-sized color molecules by the test material. Other factors such as the difference in the number of teeth in the experimental groups, the experimental conditions, and the experience of the experimenter are justifiable for these differences.

## 5. Conclusion

Based on the results of this study, we proposed that CEM cement and Biodentine with similar characteristics to MTA may be used as a coronal plug in preventing microleakage and cervical external root resorption in internal bleaching. The current study shows that CEM cement offers a better longer-term seal. Future studies with more sample size, longer duration of bleaching procedure, and clinical follow-up in patients and using SEM or confocal laser scanning microscopy for analysis marginal seal or adaptation are suggested.

Many variables could alter the microbiological composition of oral environment, such as prosthodontic frameworks [48], orthodontic appliances [49], or dental implants [50]. Therefore, the results of the present report should be confirmed with future clinical research taking into account also these variables.

## Figures and Tables

**Table 1:** Mean and median survival time of microleakage.

Groups	Mean				Median			
	Estimate	Std. error	95% confidence interval		Estimate	Std. error	95% confidence interval	
			Lower bound	Upper bound			Lower bound	Upper bound
MTA	22.000	4.287	13.598	30.402	19.000	4.961	9.277	28.723
Biodentine	15.313	4.485	6.522	24.103	3.000	7.000	0.000	16.720
CEM cement	24.000	4.950	14.298	33.702	24.000	11.906	0.664	47.336
Overall	20.438	2.699	15.147	25.728	14.000	4.944	4.309	23.691

## Data Availability

No data were used to support this study.

## References

[1] Chng H., Palamara J., Messer H. (2002). Effect of hydrogen peroxide and sodium perborate on biomechanical properties of human dentin. *Journal of Endodontics*.

[2] Plotino G., Buono L., Grande N. M., Pameijer C. H., Somma F. (2008). Nonvital tooth bleaching: a review of the literature and clinical procedures. *Journal of Endodontics*.

[3] Valera M. C., Camargo C. H. R., Carvalho C. A. T., Oliveira L. D. d., Camargo S. E. A., Rodrigues C. M. (2009). Effectiveness of carbamide peroxide and sodium perborate in non-vital discolored teeth. *Journal of Applied Oral Science*.

[4] Isola G., Polizzi A., Santonocito S., Alibrandi A., Ferlito S. (2019). Expression of salivary and serum malondialdehyde and lipid profile of patients with periodontitis and coronary heart disease. *International Journal of Molecular Sciences*.

[5] Isola G., Polizzi A., Iorio-Siciliano V., Alibrandi A., Ramaglia L., Leonardi R. (2020). Effectiveness of a nutraceutical agent in the non-surgical periodontal therapy: a randomized, controlled clinical trial. *Clinical Oral Investigations*.

[6] Isola G., Alibrandi A., Currò M. (2020). Evaluation of salivary and serum asymmetric dimethylarginine (ADMA) levels in patients with periodontal and cardiovascular disease as subclinical marker of cardiovascular risk. *Journal of Periodontology*.

[7] Zarenejad N., Asgary S., Ramazani N., Haghshenas M. R., Rafiei A., Ramazani M. (2015). Coronal micro leakage of three different dental biomaterials as intra-orifice barrier during nonvital bleaching. *Dental Research Journal (Isfahan)*.

[8] Fuss Z., Szajkis S., Tagger M. (1989). Tubular permeability to calcium hydroxide and to bleaching agents. *Journal of Endodontics*.

[9] Torabinejad M., Ung B., Kettering J. D. (1990). In vitro bacterial penetration of coronally unsealed endodontically treated teeth. *Journal of Endodontics*.

[10] Raina R., Loushine R. J., Weller R. N., Tay F. R., Pashley D. H. (2007). Evaluation of the quality of the apical seal in Resilon/Epiphany and gutta-percha/AH Plus-filled root canals by using a fluid filtration approach. *Journal of Endodontics*.

[11] Canoglu E., Gulsahi K., Sahin C., Altundasar E., Cehreli Z. (2012). Effect of bleaching agents on sealing properties of different intraorifice barriers and root filling materials. *Medicina Oral Patología Oral Y Cirugia Bucal*.

[12] Dietschi D. (2006). Nonvital bleaching: general considerations and report of two failure cases. *The European Journal of Esthetic Dentistry: Official Journal of the European Academy of Esthetic Dentistry*.

[13] Mohammadi Z., Khademi A. (2006). An evaluation of mta cements as coronal barrier. *Iranian Endodontic Journal*.

[14] Barrieshi-Nusair K. M., Hammad H. M. (2005). Intracoronal sealing comparison of mineral trioxide aggregate and glass ionomer. *Quintessence International*.

[15] Torabinejad M., Chivian N. (1999). Clinical applications of mineral trioxide aggregate. *Journal of Endodontics*.

[16] Mah T., Basrani B., Santos J. (2003). Periapical inflammation affecting coronally-inoculated dog teeth with root fillings augmented by white MTA orifice plugs. *Journal of Endodontics*.

[17] Cummings G. R., Torabinejad M. (1995). RS 53 Mineral trioxide aggregate (MTA) as an isolating barrier for internal bleaching. *Journal of Endodontics*.

[18] Tselink M., Baumgartner J. C., Marshall J. G. (2004). Bacterial leakage with mineral trioxide aggregate or a resin-modified glass ionomer used as a coronal barrier. *Journal of Endodontics*.

[19] Parirokh M., Torabinejad M. (2010). Mineral trioxide aggregate: a comprehensive literature review-Part III: clinical applications, drawbacks, and mechanism of action. *Journal of Endodontics*.

[20] Zarean P., Roozbeh R., Zarean P., Zare Jahromi M., Mirzakoochaki Broujeni P. (2019). In vitro comparison of shear bond strength of a flowable composite resin and a single-component glass-ionomer to three different pulp-capping agents. *Dental and Medical Problems*.

[21] Mozayeni M. A., Milani A. S., Marvasti L. A., Asgary S. (2012). Cytotoxicity of calcium enriched mixture cement compared with mineral trioxide aggregate and intermediate restorative material. *Australian Endodontic Journal*.

[22] Mamak A., Moradi Majd N., Shivaie Kojoori S., Norooz Oliaie H., Naghavi N., Asgary S. (2012). Comparison of endodontic biomaterials as apical barriers in simulated open apices. *ISRN Dentistry*.

[23] Grech L., Mallia B., Camilleri J. (2013). Characterization of set intermediate restorative material, biodentine, bioaggregate and a prototype calcium silicate cement for use as root-end filling materials. *International Endodontic Journal*.

[24] Han L., Okiji T. (2013). Bioactivity evaluation of three calcium silicate-based endodontic materials. *International Endodontic Journal*.

[25] Zhou H.-M., Shen Y., Wang Z.-J. (2013). In vitro cytotoxicity evaluation of a novel root repair material. *Journal of Endodontics*.

[26] Aksel H., Küçükkaya Eren S., Askerbeyli Õrs S., Karaismailoğlu E. (2019). Surface and vertical dimensional changes of mineral trioxide aggregate and biodentine in different environmental conditions. *Journal of Applied Oral Science*.

[27] Rajasekharan S., Martens L. C., Cauwels R. G. E. C., Verbeeck R. M. H. (2014). Biodentine material characteristics and clinical applications: a review of the literature. *European Archives of Paediatric Dentistry*.

[28] Allan N., Walton R., Schaffer M. (2001). Setting times for endodontic sealers under clinical usage and in vitro conditions. *Journal of Endodontics*.

[29] Cohen S., Burns R. C. (2002). *Pathways of the Pulp*.

[30] Hargreaves K., Berman L. (2016). *Cohen's Pathways of the Pulp*.

[31] Santos L. G. P. d., Felippe W. T., Souza B. D. M. d., Konrath A. C., Cordeiro M. M. R., Felippe M. C. S. (2017). Crown discoloration promoted by materials used in regenerative endodontic procedures and effect of dental bleaching: spectrophotometric analysis. *Journal of Applied Oral Science*.

[32] Moradi S., Disfani R., Ghazvini K., Lomee M. (2013). Sealing ability of orthograde MTA and CEM cement in apically resected roots using bacterial leakage method. *Iranian Endodontic Journal*.

[33] Timpawat S., Amornchat C., Trisuwan W. (2001). Bacterial coronal leakage after obturation with three root canal sealers. *Journal of Endodontics*.

[34] Han L., Okiji T. (2011). Uptake of calcium and silicon released from calcium silicate-based endodontic materials into root canal dentine. *International Endodontic Journal*.

[35] Veríssimo D. M., do Vale M. S. (2006). Methodologies for assessment of apical and coronal leakage of endodontic filling materials: a critical review. *Journal of Oral Science*.

[36] Britto L. R., Grimaudo N. J., Vertucci F. J. (2003). Coronal microleakage assessed by polymicrobial markers. *The Journal of Contemporary Dental Practice*.

[37] Navabi A. A., Khademi A. A., Khabiri M., Zarean P., Zarean P. (2018). Comparative evaluation of *Enterococcus faecalis* counts in different tapers of rotary system and irrigation fluids: an *ex vivo* study. *Journal of Dental Research*.

[38] Estrela C., Bammann L. L., Estrela C. R., Silva R. S, Pécora J. D (2000). Antimicrobial and chemical study of MTA, Portland cement, calcium hydroxide paste, sealapex and dycal. *Brazilian Dental Journal*.

[39] Bhavana V., Chaitanya K., Dola B., Gandi P., Patil J., Reddy R. (2015). Evaluation of antibacterial and antifungal activity of new calcium-based cement (Biodentine) compared to MTA and glass ionomer cement. *Journal of Conservative Dentistry*.

[40] Jose J., K S., Tomy N., Aman S. (2016). Comparative evaluation of antimicrobial activity of biodentine and MTA against e. faecalis an in vitro study. *International Journal of Dental Research*.

[41] Nourzadeh M., Amini A., Fakoor F., Asgary S. (2019). Antimicrobial activity of calcium-enriched mixture cement and biodentine on Enterococcus faecalis: an in vitro study. *Iranian Endodontic Journal*.

[42] Razmi H., Shokouhinejad N., Fekrazad R., Motahhary P., Alidoust M. (2009). Comparison of the sealing ability of two root-end filling materials (MTA and CEM cement) following retropreparation with ultrasonic or Er, Cr: YSGG laser. *Journal of Dental Medicine*.

[43] Sarkar N., Caicedo R., Ritwik P., Moiseyeva R., Kawashima I. (2005). Physicochemical basis of the biologic properties of mineral trioxide aggregate. *Journal of Endodontics*.

[44] Moghadam N., Abdollahi A. A., Aghabalayi Fakhim H., Borna Z. (2017). In vitro sealing properties of calcium-enriched mixture and mineral trioxide aggregate orifice barriers during intra-coronal bleaching. *Iranian Endodontic Journal*.

[45] Ramazani N., Sadeghi P. (2016). Bacterial leakage of mineral trioxide aggregate, calcium-enriched mixture and biodentine as furcation perforation repair materials in primary molars. *Iranian Endodontic Journal*.

[46] Bolhari B., Ashofteh Yazdi K., Sharifi F., Pirmoazen S. (2015). Comparative scanning electron microscopic study of the marginal adaptation of four root-end filling materials in presence and absence of blood. *Journal of dentistry (Tehran)*.

[47] Ramezanali F., Aryanezhad S., Mohammadian F., Dibaji F., Kharazifard M. J. (2017). In vitro microleakage of mineral trioxide aggregate, calcium-enriched mixture cement and biodentine intra-orifice barriers. *Iranian Endodontic Journal*.

[48] Stencel R., Kasperski J., Pakieła W. (2018). Properties of experimental dental composites containing antibacterial silver-releasing filler. *Materials (Basel)*.

[49] Sfondrini M. F., Debiaggi M., Zara F. (2012). Influence of lingual bracket position on microbial and periodontal parameters in vivo. *Journal of Applied Oral Science*.

[50] Alagl A. S., Madi M., Bedi S., Al Onaizan F., Al-Aql Z. S. (2019). The effect of Er,Cr:YSGG and diode laser applications on dental implant surfaces contaminated with acinetobacter baumannii and Pseudomonas aeruginosa. *Materials (Basel)*.

